# A Recurrent Neural Network for Attenuating Non-cognitive Components of Pupil Dynamics

**DOI:** 10.3389/fpsyg.2021.604522

**Published:** 2021-02-01

**Authors:** Sharath Koorathota, Kaveri Thakoor, Linbi Hong, Yaoli Mao, Patrick Adelman, Paul Sajda

**Affiliations:** ^1^Department of Biomedical Engineering, Columbia University, New York, NY, United States; ^2^Fovea Inc., New York, NY, United States; ^3^Department of Cognitive Science, Columbia University, New York, NY, United States

**Keywords:** recurrent neural network, pupil diameter, eye tracking, video viewing, pupil response

## Abstract

There is increasing interest in how the pupil dynamics of the eye reflect underlying cognitive processes and brain states. Problematic, however, is that pupil changes can be due to non-cognitive factors, for example luminance changes in the environment, accommodation and movement. In this paper we consider how by modeling the response of the pupil in real-world environments we can capture the non-cognitive related changes and remove these to extract a residual signal which is a better index of cognition and performance. Specifically, we utilize sequence measures such as fixation position, duration, saccades, and blink-related information as inputs to a deep recurrent neural network (RNN) model for predicting subsequent pupil diameter. We build and evaluate the model for a task where subjects are watching educational videos and subsequently asked questions based on the content. Compared to commonly-used models for this task, the RNN had the lowest errors rates in predicting subsequent pupil dilation given sequence data. Most importantly was how the model output related to subjects' cognitive performance as assessed by a post-viewing test. Consistent with our hypothesis that the model captures non-cognitive pupil dynamics, we found (1) the model's root-mean square error was less for lower performing subjects than for those having better performance on the post-viewing test, (2) the residuals of the RNN (LSTM) model had the highest correlation with subject post-viewing test scores and (3) the residuals had the highest discriminability (assessed via area under the ROC curve, AUC) for classifying high and low test performers, compared to the true pupil size or the RNN model predictions. This suggests that deep learning sequence models may be good for separating components of pupil responses that are linked to luminance and accommodation from those that are linked to cognition and arousal.

## 1. Introduction

### 1.1. Pupillary Response

Physiological measures during cognitive processing have been extensively studied with pupillary dilation, in particular, having been explored as an index of learning, cognitive load, attention and memory (Sibley et al., 2011; Wang, 2011; Fridman et al., 2018). Dilation is generally understood to be mediated by increased sympathetic activity or inhibition of the parasympathetic response (Karatekin, 2007) and reflected by activity in the brain's locus coeruleus-norepinephrine system (LC-NE), which controls physiological arousal and attention. LC-NE activity has been correlated with subjective task difficulty, cognitive effort, and neural gain (Eckstein et al., 2017). Mechanistically, the responsiveness of the pupil is driven by antagonistic actions of the iris dilator and sphincter muscles (Joos and Melson, 2012). Specific cognitive influences include pupil dilation in response to error in risk prediction and decision making (de Gee et al., 2014; Buettner et al., 2018), to emotional arousal (Hess, 1972), and in the presence of a known visual target (Privitera et al., 2010). In addition, the pupil has been shown to dilate to increased processing load in language tasks (Wang, 2011).

Pupil dilation is also important for regulating light entering the eye (Winn et al., 1994) and thus measures of cognitive processes linked to the pupil are confounded by: (1) the natural dilation changes due to luminance, (2) the photometric measure of light entering the eye, or (3) accommodation, the process by which the eye keeps focus on an object across varying distances. It is established that the pupil constricts with increasing luminance (Raiturkar et al., 2016), as the former is modulated by the pretectal nucleus. In fact, multiple studies have shown that luminance conditions take priority over cognitive demands in pupil diameter changes, across task difficulty and modality (Xu et al., 2011; Kun et al., 2012; Peysakhovich et al., 2015). Accommodation also effects pupil diameter to a lesser extent and appears to be limited as a driver in younger populations (Mathur et al., 2014).

### 1.2. Learning and Eye Tracking

In addition to pupillary response reflecting cognitive processing, past work has examined how other eye movements, such as fixations, can be indicators of cognitive processing when viewing educational content. Eye movements are more variable and less restricted by content boundaries in a younger audience while viewing Sesame Street, and video comprehension increases with age (Kirkorian et al., 2012). As visual and auditory saliency has strong direct impacts on visual exploration (which is captured by eye movement) and therefore indirect impacts on learning (Coutrot et al., 2014), eye movement information can be used to predict subjects' attention to viewing content.

The use of eye tracking data to help understand how students process content derived from different modalities has been employed to study how attention on PowerPoint slides changes with or without relevant narration (Slykhuis et al., 2005). Furthermore, viewing behavior has been used to assist in prediction of learning styles, using post-viewing assessments and viewing ratios (Cao and Nishihara, 2012) and, more recently, gaze behaviors such as fixations have been shown to vary with perceived relevance and presentation modalities of instructional content (Wiedbusch and Azevedo, 2020).

Simple eye tracking models have been employed to predict attention using measures such as total fixation duration (Xu et al., 2008). In our case, we seek to model how the input space predicts pupil dilation, using fixational and pupil features from eye tracking data along with contextual features from instructional video. While pupil dilation is most strongly affected by luminance-driven changes, recent work has yielded encouraging results in using pupil diameter to track lapses in attention (van den Brink et al., 2016), cognitive load (Wang, 2011) and as an index of learning (Sibley et al., 2011). One possible approach to distinguish between attention and luminance-driven effects is through comparison of model accuracy between above- and below-average performers in learning tasks. We hypothesize that in such a comparison, pupil diameter will be more variable and thus harder to predict in above-average performers, who may be more driven by pupil-linked arousal fluctuations.

### 1.3. Modeling Eye Tracking Data

To detect eye tracking events of interest, random forest models have previously been employed to detect fixations, saccades, and post-saccadic oscillations, yielding close-to-human level annotations (Buettner et al., 2018). Visual attention modeling has utilized video-level features, mapping these features to spatial and temporal saliency maps (Fang et al., 2017) in order to model gaze preferences. Bayesian networks and hidden Markov models have been used to learn patterns in eye movements to recognize facial expressions (Bagci et al., 2004; Datcu and Rothkrantz, 2004). Recent work has also analyzed still video frames through convolutional neural networks to analyze gaze data with the purpose of classifying groups (Dalrymple et al., 2019). However, sequences of fixations over areas of interest may also be useful in distinguishing individuals and groups (Çöltekin et al., 2010). In general, linear models, including those that employ regularized regression (ridge and lasso) (Papoutsaki et al., 2016) are simple and typically less likely to overfit the data. Non-linear models, including recurrent neural networks (RNNs) are interesting to consider as an alternative to linear models. For example, though RNNs are more complex and typically have more parameters then their linear counterparts, they can learn state sequence information over multiple timescales and feature dimensions. The long short-term memory model (LSTM) is a form of recurrent neural network that learns parameters over large amounts of sequence data efficiently (Hochreiter and Schmidhuber, 1997). LSTMs are used in language modeling, for example, as they are particularly suited to sequence data, and have been shown to outperform traditional deep learning network architectures (Sundermeyer et al., 2012; Koorathota et al., 2020). Because of this, the use of a sequence model such as an LSTM is a natural next step in analyzing gaze sequences.

### 1.4. The Present Study

The primary aim of the this study was to assess the prediction of pupil diameter in groups of participants whose performance varied on post-viewing assessments of educational content. We hypothesize that, due to the viewing dynamics, the realistic content, and the fact that information conveyed in the video is sparse compared to the length of the videos, a model that predicts pupil dynamics will tend to learn non-cognitive components, e.g., dynamics due to luminance changes, motion, accommodation. In this case we expect the residuals of the pupil dynamics under the model, i.e., those dynamics which are not predictable by the model, to be more informative of cognitive performance.

Toward that end, we initially compared accuracy of linear, non-linear, and RNNs when predicting pupil diameter. We further varied the type of input features we used as input to our models, to parse the usefulness of various eye movements and events when predicting pupil diameter. We then correlated the residuals from the most accurate models with performance on the post-viewing assessments to understand how accuracy of prediction varies across performers. We found that, compared to other models, the RNN (LSTM) (1) had root-mean square error (RMSE) that was less for lower performing subjects than for those having better performance on the post-viewing test, (2) the residuals of the model had the highest correlation with subject post-viewing test scores and (3) the residuals had the highest discriminability (assessed via area under the ROC curve, AUC) for classifying high and low test performers.

## 2. Methods Summary

### 2.1. Study Summary

61 healthy subjects (47 female, ages 18–35 with a mean of 25) participated in this study. Informed consent was obtained from all volunteers and the Columbia University Institutional Review Board approved all experiments. Participants were randomly assigned into three modality conditions to watch three 5-min-long lecture videos, with their eye movements recorded. After each video, they were instructed to answer a set of 7 multiple-choice questions, with a single correct answer, assessing comprehension of the video content just shown.

The lecture videos consisted of slides with images and bullet-point lists, presented by a professor in an academic classroom setting. Videos were produced to closely mimic the type of lecture students were likely to encounter in a real-life college-level academic setting as well as to provide sufficient context so that no subject-specific familiarity and expertise with the topic is required to answer the questions. The specific selection criteria for the lectures were as follows:

1. They had to be complex in content and be on topics that the participants were unlikely to be very familiar with but were also likely to find interesting,
2. They had to have visuo-spatial content that would allow for both images and a diverse set of gestures.

**Figure 1:**
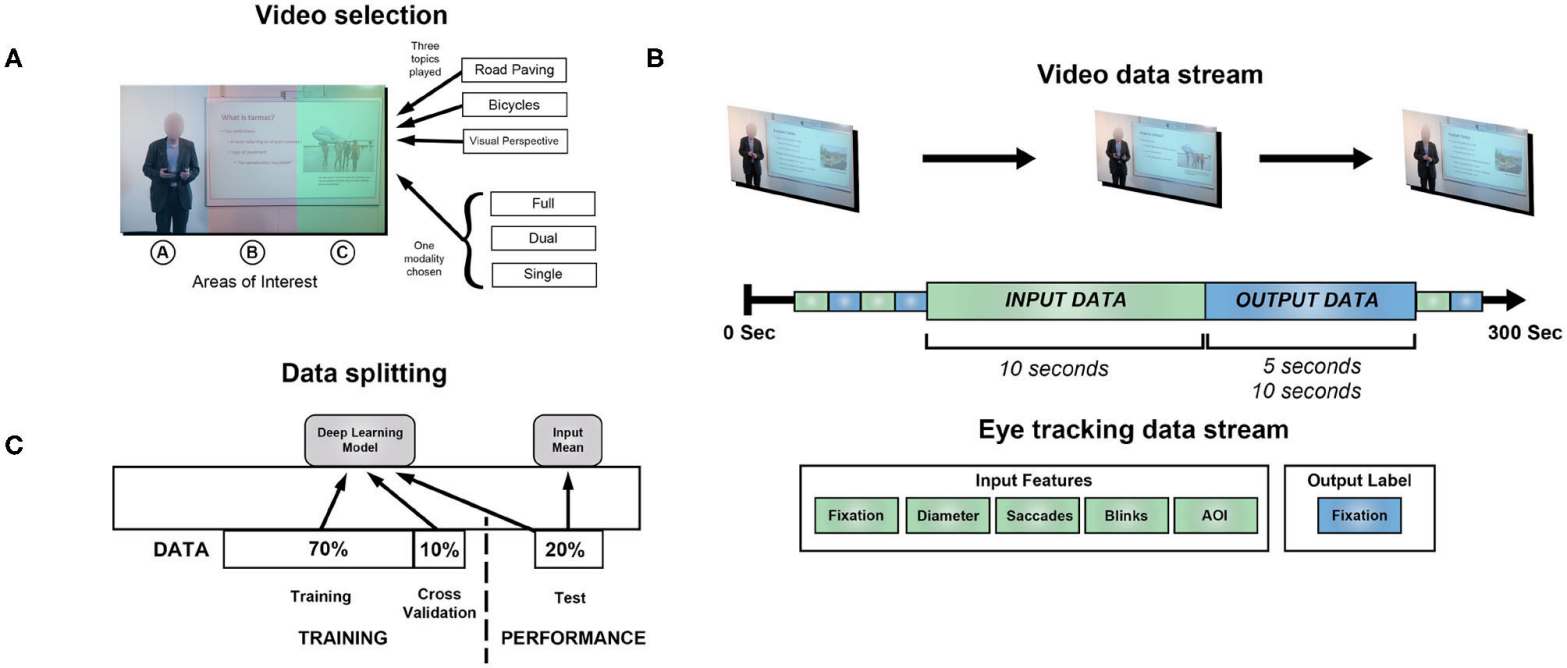
Overview of methods. **(A)** Participants viewed videos on three topics while eye tracking data was collected: visual perspective, bicycles, and road paving. They were randomized to one of three modalities: full, dual or single. Colorized in the image are the areas of interest (AOI) used in training several of the models we consider. Subjects did not see this colorization nor were they explicitly aware of the AOIs. **(B)** The video spanned 300 s, while the eye tracking data was split into 10 or 15 s blocks over the course of viewing. The first 10 s was used as input data toward the model, with various types of features. **(C)** The blocks of eye tracking data were split into training, validation and test data for model fitting and testing. Predictions were derived from the model frameworks, e.g., the deep learning model, and the mean of the input pupil diameter, for a naive estimate.

We chose the following three topics: the history of tarmac road paving, the use of perspectives in drawing, and the history of bicycles (Figure 1A). Additionally, speaker style and movement, as well as video editing techniques (cuts, edits, graphics, and sound effects) were also controlled in the video production using pre-specified scripts.

Of the questions assessing comprehension, 6 were slide-specific, in that the information used to answer each question was contained in one slide, and the remaining question required information across the presentation. The validity of questions were tested in a pilot study with 7 additional subjects so that ambiguous or unclear wording was clarified and items too difficult or easy were revised to have the proper discriminability to evaluate understanding.

The three modality conditions (i.e., video types) were produced with the same audio content but different types of visual content, including single (full-screen slides), dual (slides and audio lecture), and full (professor with upper body view visible on the lecture video, with slides present) versions. Using a between-subject design, each subject was shown the same modality version for all three topics—controlling for luminance across viewing sessions. The topics were always presented in the same order: history of road paving, visual perspectives, and history of bicycles.

### 2.2. Eye Movements and Pupil Dilation

Eye tracking was performed with an Eyelink 1000 in Tower Mount, at a sampling rate of 1 kHz. Eye tracking data contained X and Y coordinates of each fixation (pixels), fixation duration (ms), pupil diameter (μm), saccades, blinks and associated timestamps (Figure 1B).

Subjects were instructed to watch videos presented on a 30-inch screen from 40 inches away without wearing glasses. The study was conducted in a Faraday's cage with low-light, sound-proof conditions that remained constant during video watching. Before each of the three videos, the eye tracker was calibrated for each recruited subject. In the calibration procedure, subjects were asked to focus their gaze on nine points presented consecutively at specific positions across the diagonals and centers of the side edges of the display screen. Moreover, subjects were instructed not to move their heads and to pay attention to the lecture content presented on the screen throughout video watching.

For each subject, we filtered for fixations out of the video frame boundary and systematic drifts. 3 participants were found to spend a non-negligible amount of time (>6%) blinking or fixating outside of the center rectangle video frame boundary and were excluded as outliers, leaving a total of 58 subjects for further analysis.

Classifications of eye events, including fixations, saccades, and blinks were exported from the SR Research software, which uses video-oculography based classification algorithms and pupil diameter calculations.

### 2.3. Problem Types

The prediction problems or inputs varied across two dimensions: (1) the amount of time, relative to the input, used in the generation of the output label and (2) the types of input features used for predictions.

We utilized five categories of input features for the models:

- Fixations: positions, durations, start times, and respective differences from fixation to fixation,
- Pupil diameter: per fixation,
- Areas of interest: a mapping of sequence of AOI to 50-dimensional embeddings learned during training process,
- Saccades: saccade-related positions, durations, start times and respective differences,
- Blinks: blink times and differences.

We investigated the effect of various combinations of the types of inputs: {fixations, fixations + pupil diameter, fixations + saccades + blinks fixations + pupil diameter + saccades + blinks}. Because eye tracking data can be sourced from web cameras, infrared devices, or human annotations, each with varying level of accuracy for labeling eye movements and events, our aim was to assess the minimal amount of data that yields accurate predictions of pupil diameter. We were not able to find similar iterative approaches to predicting pupil diameter using different types of input data and hypothesized historical fixation and input pupil diameter to be the best predictors of future pupil diameter.

In addition, for baseline reference, we report the error rates in models that are most commonly used toward prediction of eye tracking data:

- Linear regression: simple linear fit of input features,
- Regularized regression: linear regression with penalization of large weights through L1 (Lasso) and L2 (Ridge) norms,
- Decision-tree based: ensemble learning methods relying on majority vote by weak models (gradient boosting) or mean of trees (random forest),
- Input mean: a naive estimate of the mean pupil diameter in the input.

Hyperparameters for the reference models were selected from default recommendations from scikit-learn, a popular machine learning framework in Python (Pedregosa et al., 2011).

### 2.4. Data Aggregation

Because this study was supplementary to a larger one focusing on the effects of gestures on learning, we were presented the option to use data from single or multiple modalities. The justification for using all available modalities for prediction of pupil diameter was twofold: to allow for a large enough amount of data to utilize deep learning models that we predicted would perform well, and to increase the robustness of prediction of pupil diameter under different modalities of learning. Because, in a natural learning environment, students may be presented with video and audio but may not necessarily attend to it (Chen and Wu, 2015), this dataset provided a unique opportunity to predict pupil diameter and assess model accuracy under mixed modalities.

As a first step for analysis, eye tracking streams were split into 15-s blocks, across all participants, modalities and topics, and randomized. The first 10 s in each block were used to sequence input data, while pupil diameters in fixations in the succeeding 5 s of the block were averaged to yield the associated output label. In another method of analysis, eye tracking streams were split into 20-s blocks, with features collected over the first 10 s as input and the succeeding 10-s fixation pupil diameter as output.

Subsequent analyses are reported from the best-performing model using 10 s of input to predict 5 s of output. We made this selection in order to maximize the number of samples and use typical output durations studied in past eye fixation work (Just and Carpenter, 1976).

Due to this method of data aggregation, the number of fixations, saccades and blinks varied across and within participants. Thus, the input region required feature-specific, mean padding to the maximum length of fixations. The output was always a single-dimensional, average, fixation pupil diameter gathered from the output region. Thus, the deep learning models can be thought of as regression problems utilizing a non-linear framework.

### 2.5. AOI Embeddings

We defined three, distinct, areas of interest (AOI) in the full video type, across all topics, corresponding to the instructor, text in slides and images in slides. Other types (dual and single) contained only text and image AOI.

**Figure 2:**
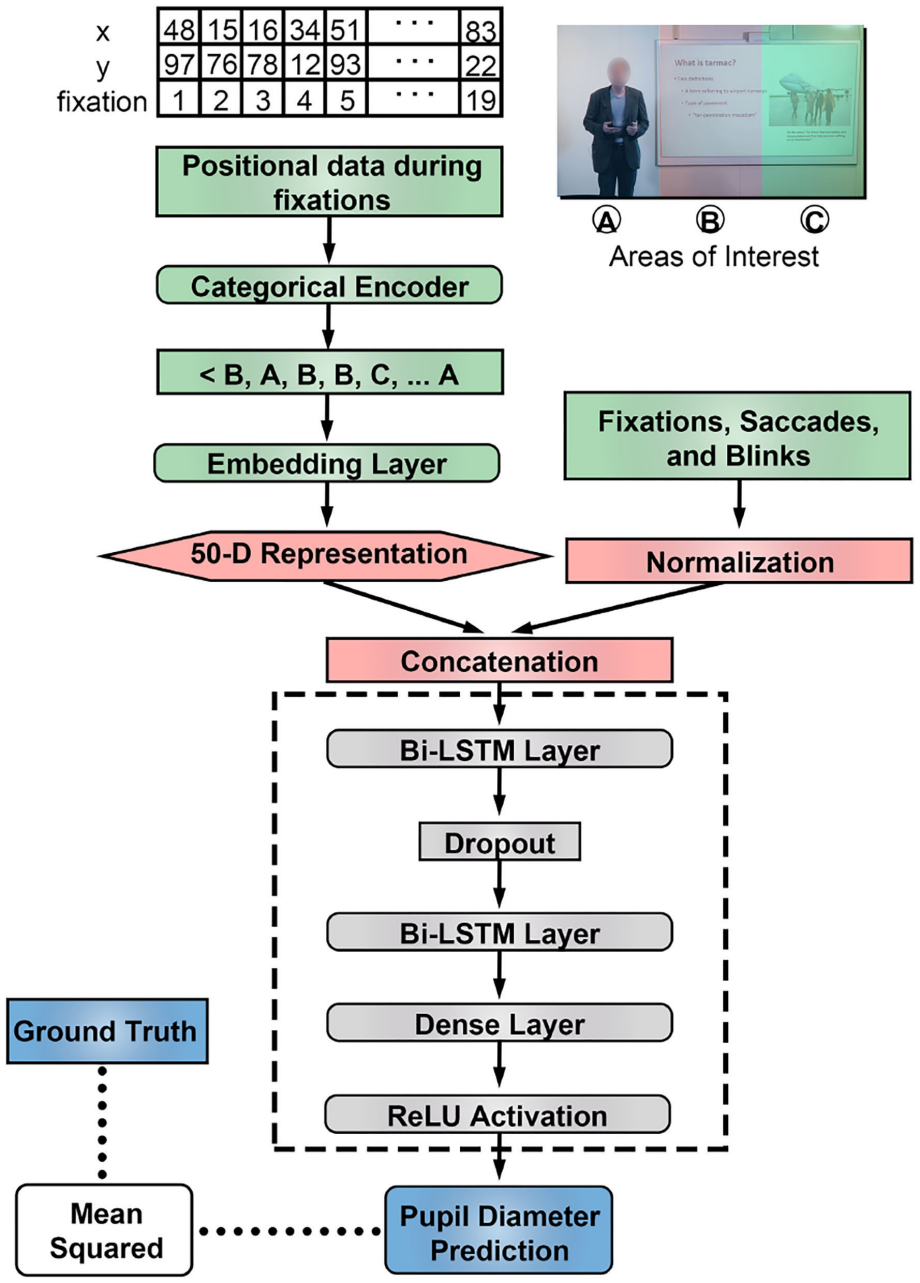
LSTM architecture included two bidirectional layers as the core component. Numerical features were normalized and the areas of interest were embedded to a higher dimensional vector trained using the training samples. Embeddings are trained using categorical representations of fixation data.

We mapped X and Y coordinates from fixations in input regions to AOI. This allowed us to generate a sequence of AOIs for fixations during a specified input region, which we used to train 50-dimensional embeddings during the training process (Figure 2). We hypothesized that this process will achieve a similar goal as in natural language applications of capturing context of categorical information with respect to other input features (Melamud et al., 2016).

### 2.6. Network Architecture

We used a bi-directional LSTM network to model eye tracking input (Figure 2). For each problem type, data was split into training, validation and test samples (Figure 1C). The network was trained and validated on estimates of pupil diameter and assessed through mean-squared error using the Adam optimizer (Table 1). Each LSTM layer used recurrent dropout to prevent overfitting.

**Table 1:** Network hyperparameters.

**Param**	**Value**
Epochs	5,000
Early stopping	500 epochs (loss)
Optimizer	Adam
Learning rate	0.0001
Training split	70%
Validation split	10%
Test split	20%

We compared our network's results to the mean pupil diameter as calculated from the fixations in the input region and with other, reported linear and non-linear approaches. In addition, we compared our LSTM network results to a gated recurrent unit (GRU), an RNN variant (Chung et al., 2014), with the same network hyperparameters and without recurrent dropout. Neural models were implemented in Tensorflow 2.2 on Google Cloud and trained using a NVIDIA Tesla K80 GPU.

### 2.7. Data Analysis

We hypothesized that predictability of pupil diameter would vary across four dimensions: (1) as a ratio of input time (used in the aggregation of input features) and output time (used to calculate for ground truth pupil diameter), (2) use of different physiological measures in the network, (3) addition of AOI embeddings in the neural network, and (4) participant performance on the post-viewing assessments.

To test the hypothesis that predictability varied across the four dimensions, we first split the data into 15-s blocks. We designed a baseline comparison through averaging the pupil diameter across fixations during the first 10 s of each block. This served as the naive, input mean, estimate.

Using the first 10 s in each block to aggregate input features, we randomly separated the data into training, validation and test sets, calculated the RMSE to study the prediction errors in the test set. We repeated the process a total of 10 times (i.e., runs) for each problem type and using different input features in the best-performing model to account for variability of accuracy due to the training and test separation of the data. Furthermore, we repeated the process above after separately splitting the data into 20-s blocks first, predicting 10 s of output. We summarize the reported RMSE measure

$$RMSE_{M,I,O}=\frac{1}{l}\overset{l}{\underset{t=1}{\sum }}\left(\sqrt{\frac{1}{n}\overset{n}{\underset{s=1}{\sum }}{\left({ŷ}_{s}-y_{s}\right)}^{2}}\right),$$

where ŷ_*s*_ is the predicted pupil diameter, *y*_*s*_ is the ground truth, output pupil diameter, *n* is the number of training samples, and *l* is the number of random, training, validation and test splits RMSE was averaged over (always 10). This value was calculated for each model type, *M*, for different sets of input features, *I*, and output period length *O* over which fixation pupil diameters were averaged.

The aggregation and split of the data led to reusing the same 15- or 20-s blocks across the 10 runs. These were treated as independent samples, regardless of the video type, condition or participant they originated from.

### 2.8. Participant Performance

To study model accuracy in groups with different levels of cognitive effort, we split the test blocks by mean performance on post-viewing assessments (i.e., into “Greater Than Mean” and “Lesser Than Mean” bins). We report model results separately for these groups, using a Mann–Whitney *U*-test for significant, mean differences in model errors.

Using residuals from the most accurate model, we report Spearman correlation coefficients in the test samples between the ground truth pupil diameter, the estimate from the model, residuals (ground truth minus model estimate) and performance. To assess the predictive accuracy directly, we designed a simple binary classification task using the ground truth pupil diameter, model estimate and residuals to classify participants as belonging to the lowest or highest tertile group by performance. We used an ROC analysis, which consists of a plot of the sensitivity and 1-specificity pairs that are produced as a single decision threshold is moved from the lowest (i.e., all participants classified in the lowest tertile) to the highest (i.e., all participants classified in the highest tertile) possible value (Fawcett, 2006). The area under the ROC curve (AUC) corresponds to the probability that a randomly selected participant will have been assessed by the measure (e.g., residuals) as performing better than a randomly selected participant, and varies from 0.5 (i.e., accuracy is not improved over chance) to 1.00 (i.e., perfect accuracy).

Thus, we used group-level RMSE differences to quantify how model accuracy varies with levels of cognitive effort and residuals to understand the relation between the accuracy of model predictions and participant performance.

## 3. Results

A total of 2,379, 20-s blocks and 3,249, 15-s blocks were analyzed, with an average pupil diameter of 2126.42 μm (SD = 916.04 μm) and 2134.33 μm (SD = 934.20 μm) respectively.

### 3.1. Model Comparisons

We first report the mean error metrics, averaged over 10 runs, for each model type in Table 2. The use of embeddings improves the model accuracy only for the LSTM, which also outperforms the other model types we tested in average RMSE. For the remaining results, we utilized the best performing model, the LSTM.

**Table 2:** Prediction errors (RMSE) for linear, regularized linear, decision-tree based, and RNN (GRU, LSTM) model types.

**Inputs**	**Model type**	**RMSE**
Fixation	Linear regression	>5000
+ Diameter	Ridge regression	332.72 (5.11)
+ Blinks	Gradient boosting	319.14 (12.05)
+ Saccades	Input mean	312.93 (13.32)
	GRU	300.91 (20.64)
	Lasso regression	295.10 (9.18)
	Random forest	292.79 (12.26)
	LSTM	285.65 (9.69)
Fixation	Linear regression	>5,000
+ Diameter	Ridge regression	332.35 (13.38)
+ Saccades	Gradient boosting	323.56 (13.34)
+ Blinks	Input mean	306.45 (11.13)
+ Embeddings	Lasso regression	304.34 (9.61)
	Random forest	298.05 (11.45)
	GRU	288.38 (12.30)
	LSTM	249.87 (8.65)

*Inputs from 10 s of each block was used to predict 5 s of subsequent, average, fixation pupil diameter. Input mean refers to the naive estimate of using the mean pupil diameter in the input data as a prediction of the pupil diameter*.

*Mean (SD)*.

### 3.2. Input Feature Comparisons

We report the aggregate accuracy, in terms of RMSE with respect to ground truth pupil diameter, of the LSTM models and the input mean (Table 3). When pupil diameter was used as an input, RMSE was significantly lower than the input mean model (312.93 μm). The best performing model used only fixation and pupil diameter measures as input, with 10 s of input predicting mean pupil diameter for 10 s of output. This had a mean RMSE of 252.97 μm.

**Table 3:** RMSE test accuracy for given set of input features using the LSTM framework, including a simple comparison using the mean pupil diameter across fixations.

**Input features**	**10 s input predicting 5 s output**			**10 s input predicting 10 s output**		
	**RMSE**	**Δ*RMSE_AOI_***	***n***	**RMSE**	**Δ*RMSE_AOI_***	***n***
Fixation	711.77^***^ (14.06)	7.7	650	723.45^***^ (32.06)	−15.19	476
Fixation + Saccades + Blinks	652.74^***^ (20.66)	−13.71	650	661.81^***^ (33.00)	−9.05	476
Mean Input Diameter	312.93 (13.32)	-	650	302.98 (14.19)	-	476
Fixation + Diameter + Saccades + Blinks	285.65^**^ (9.69)	−35.78^***^	650	266.27^***^ (12.65)	−9.68	476
Fixation + Diameter	270.71^***^ (10.74)	−35.12^***^	650	252.97^***^ (9.35)	−16.91^***^	476

*Metrics are reported as mean (SD) and were averaged across 10 random test splits. Differences between the input mean to other models were assessed using Mann–Whitney U-Test. Deltas indicate differences in test accuracy measures after adding AOI embeddings to models, assessed using the Mann–Whitney U-test*.

* ≤ 0.05,

** *≤ 0.01*,

*** *≤ 0.001*.

Generally, when pupil diameter was used as an input, accuracy significantly improved as output length increased from 5 to 10 s.

### 3.3. Addition of Embeddings

Next, we report the change in RMSE as a result of adding the AOI embeddings (Table 3). When using pupil diameter as an input, adding AOI embeddings significantly reduced the RMSE. In these cases, the drop in RMSE was significantly more than 35 μm, with a more pronounced effect when predicting output pupil diameter in 5 s. The effect of AOI was less pronounced when predicting pupil diameter averaged over the longer time span of 10 s, indicated by less reduced RMSE and non-significant reductions even in the condition utilizing the full set of input features (−9.68 μm, *p* > 0.05). Note, subsequent analyses is reported only for the 5 s output condition.

### 3.4. Performance Differences

The average, post-lecture, performance on the assessment was determined to be 59% across participants, video types and conditions. Thus, we report the accuracy of the LSTM and input mean models in participants who performed greater or lesser than this mean.

In all cases, model accuracy was relatively better in participants who scored below the mean (Table 4). In the best-performing case (using fixation and pupil diameter as input), the RMSE, on average, decreased by 31.13 μm (*p* < 0.01) when using the same model for below-average compared to above-average performers.

**Table 4:** Mean (SD) accuracy differences after splitting data into above- and below- average (0.59) performers on the post-viewing assessments using the LSTM framework.

**Input features**	**Greater Than Mean**				**Lesser Than Mean**		
	**RMSE**	**Δ*RMSE_AOI_***	***n***	**ΔRMSE**	**RMSE**	**Δ*RMSE_AOI_***	***n***
Fixation	736.68^***^ (34.78)	30.70	281	^**^	692.04^***^ (22.98)	−8.43	368
Fixation + Saccades + Blinks	679.21^***^ (38.70)	−7.84	284	^**^	632.6^***^ (18.46)	−18.34^*^	366
Mean input diameter	347.48 (24.60)	-	284	^**^	282.95 (10.13)	-	366
Fixation + Diameter + Saccades + Blinks	296.26^***^ (14.00)	−29.52^***^	284	^**^	277.19^***^ (11.91)	−40.71^***^	366
Fixation + Diameter	288.21^***^ (16.02)	−34.23^***^	284	^**^	257.08^***^ (15.10)	−36.00^***^	365

*Significance assessed using the Mann–Whitney U-test for mean differences in RMSE (across 10 random, test data splits or model runs) between sets of input features and mean input pupil diameter, and separately for delta scores after addition of AOI (ΔRMSE_AOI_) within groups. We also report differences in between the above- and below-average performers using various input features (Δ RMSE)*.

* *≤ 0.05*,

** *≤ 0.01*,

*** *≤ 0.001*.

The input features whose associated accuracy resulted in the greatest difference between groups, surprisingly, was the input mean pupil diameter, showing a significant difference of 64.53 μm (*p* < 0.01) between below- and above-average performers. All other frameworks, using different input features, experienced better prediction in the below-average performers (*p* < 0.01).

We found a similar pattern of reduction as in the case of aggregate analysis (Table 3) in RMSE after adding in AOI embeddings for both above- and below-average performers.

**Figure 3:**
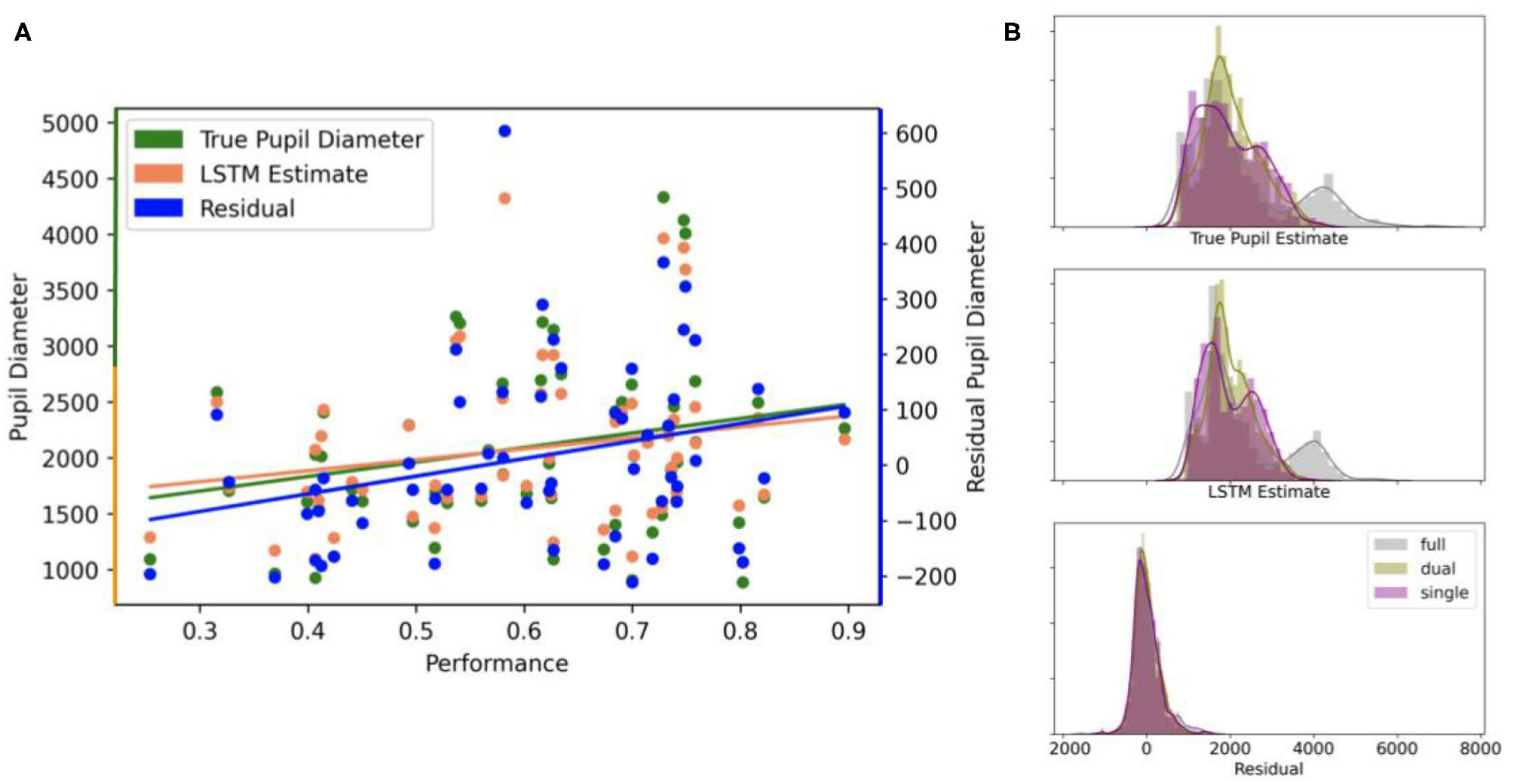
**(A)** Correlational relationships in test samples, averaged by participant, between the true (ground truth) pupil diameter, the estimate from the LSTM model, and the residual (ground truth minus model estimate) with participant performance. True pupil diameter (green) and the LSTM estimate (orange) values use the left axis scale, while residual values (blue) use the right axis scale. **(B)** Distributions of true pupil diameter, LSTM estimates, and residuals by video modality.

We also computed the correlation between ground truth, estimated, and residual (ground truth minus estimate) pupil diameter with participant performance (Figure 3A). Performance correlated significantly (at the 0.01 significance level) with the residuals from the LSTM model (*r* = 0.33), but not the true pupil diameter (*r* = 0.24) or the LSTM estimate (*r* = 0.21) at the 0.05 level. A Fisher *Z*-test showed that the difference between the correlations derived from the residuals and true pupil diameter were not significantly different at the 0.05 level (*z* = 0.66). We plot the distributions, by modality, of the true pupil diameter (mean ± SD): 2285.57 ± 1237.60 μm full, 2024.77 ± 677.94 μm dual, 1981.92 ± 758.19 μm single; LSTM estimate: 2252.86 ± 1031.46 μm full, 2018.38 ± 544.00 μm dual, 1979.03 ± 609.66 μm single; and residual: 32.72 ± 357.61 μm full, 6.39 ± 271.39 μm dual, 2.89 ± 315.49 μm single in the test samples (Figure 3B). Interesting to note is that the residuals of the model are more invariant to the variations in modality type, then the actual pupil measures or the models predictions. This is likely to reflect variation in non-cognitive measures across modality that are captured by the model and are attenuated in the residuals.

**Figure 4:**
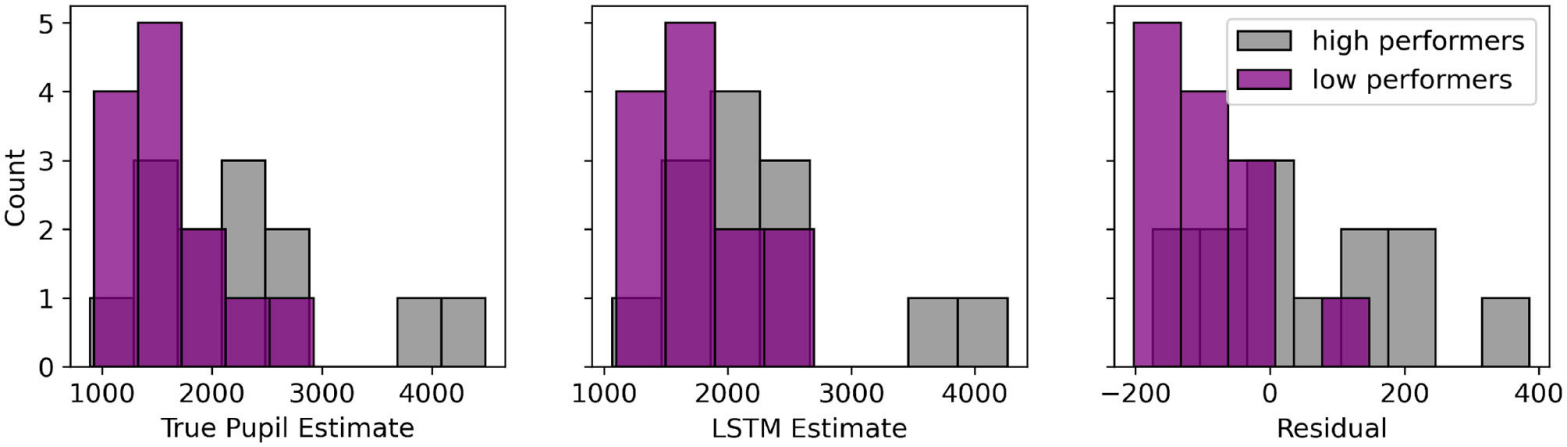
Histograms of true pupil diameter, LSTM estimates and residuals of the bottom (purple, *n* = 13) and top (gray, *n* = 13) tertile of participant performance on post-viewing assessments. The AUC is calculated for each measure. (*AUC*_*residuals*_ = 0.74, *AUC*_*LSTM*_ = 0.63, *AUC*_*pupil*_ = 0.65).

As a further analysis, we computed the separation between performance group classes (i.e., highest and lowest tertile of mean post-viewing test scores) using AUC measures (see Figure 4). AUC was largest for the model residuals compared to the model prediction and true pupil diameter measurements (*AUC*_*residuals*_ = 0.74, *AUC*_*LSTM*_ = 0.63, *AUC*_*pupil*_ = 0.65). To construct a null for significance testing, we performed 10, 000 permutations of class labels and found residuals-derived AUC (*p* < 0.01) and true pupil diameter-derived AUC (*p* = 0.05) were significantly greater than chance while the model prediction-derived AUC was not. This provides further evidence that the residuals of the model are informative of cognitive performance.

## 4. Discussion

Using viewing instructional video as a test case, we found that an LSTM recurrent network was able to indirectly disentangle luminance and cognitive processes that affect pupil dilation. The effect is indirect in that the LSTM appears to better model non-cognitive components of the pupil dynamics. For example we see higher RMSE for subjects performing better on the post-lecture assessments, while conversely, lower RMSE for those performing less well.

Since the model was trained just to predict pupil response and not cognitive effort, it is reasonable to assume most of the pupil dynamics will be attributable to non-cognitive factors given the information presented in the video is temporally sparse relative to the length of the video. Thus, under our assumptions that:

1. Higher performance in the post-viewing assessments correlates with increased cognitive performance or effort and
2. Cognitive effort is more difficult to model than lower-level drivers of pupil diameters like luminance,

we believe our sequence networks are modeling changes in the pupil dilation that reflect luminance changes, and thus model the confound that researchers often try to control for when studying attention through eye tracking data.

This finding is strengthened by the significance of correlation between LSTM residuals and performance. The LSTM thus may act as a filter to attenuate non-cognitive information in the pupil dynamics, with the residuals of the resulting signal reflecting cognitive components of the pupillary response. AUC measures followed similar trends, with a simple, binary classifier yielding better accuracy in separating performance groups using the residuals over the true pupil diameter and LSTM estimates. We recommend future paradigms use more extensive assessments to improve statistical power in related tasks.

### 4.1. Pupil Diameter Prediction

Under constant, 15.9 lux ambient illumination, pupil sizes for males and females aged 19 have been reported to vary around a mean of approximately 7,100 μm by 900 μm (one SD) (MacLachlan and Howland, 2002). Given this fact, even the simple, input mean is a reasonable predictor of pupil diameter during video viewing (Table 3). However, the best performing model (LSTM using fixation + diameter + saccades + blinks + AOI) provides a much more narrow estimate (235.59 μm) of pupil diameter across all participants. We attribute this increased accuracy to the non-linear learning capability of LSTMs, which appear to successfully learn relationships between the input features and, especially using the contextual information stored in AOI embeddings, predict pupil diameter with relatively low error in the test sets. While the GRU counterpart also had reduced prediction errors relative to the linear models, we note that the average RMSE was greater than the LSTM, and the variability in performance was larger. Furthermore, the GRU model performs worse, relative to the LSTM, when AOI embeddings are not used as input (Table 2). This may be due to the relatively increased control that the LSTM network architecture provides, which in this case may have improved the modeling of input eye events. In fact, this finding is consistent with existing literature showing RNN results vary with the complexity of sequences in a dataset (Chung et al., 2014).

Other non-linear models we evaluated for prediction of pupil diameter included random forests and gradient boosted regression trees. We hypothesized, due to the aforementioned benefits of non-linear models, that errors would be reduced for non-linear models compared to their linear counterparts. This was generally true, but the linear methods with regularization (i.e., Lasso and Ridge Regression) were similar in their error rates to non-linear methods.

We interpret the findings from reduced error rates using recurrent methods, relative to the naive, input mean estimate, to support the view that temporal memory is critical for accurate prediction of pupil diameter using eye tracking data. This accurate prediction may provide more opportunities for human-computer interaction through inferring cognitive state (Medathati et al., 2020). While, our videos' intrinsic characteristics (e.g., luminance, hue) may be highly correlated with video AOIs and this may extend to correlation with pupil diameter for bottom-up processes that rely on stimuli saliency, we believe this extension complements the goal of our study. In fact, we train our models on data from multiple modalities for this reason precisely—because we believe that a video's intrinsic characteristics might be confounds for pupil dynamics and not assessment performance, and modeling approaches may work better for saliency-driven pupil changes and not cognitively-driven changes.

### 4.2. Improvement From AOIs

We fixed AOIs to be constant across videos, since we wanted to isolate regions most relevant to information processing in the given task. By controlling where and how information is presented in the videos, we attempted to study the effect of information presentation (e.g., through controlled text placements and instructor gestures) on pupil diameter. Our sequence model approach generally worked best when including not just eye tracking features but also context (via AOI embeddings). In all cases, adding AOI reduces RMSE—significantly in cases where pupil diameter is used as an input. Our findings indicate that pupil diameter, paired with fixational positions, provide a richer context of viewing patterns that allow accurate predictions of pupil diameter. We found a greater decrease in error when adding AOI embeddings as input predicting 5 s of average fixation pupil diameter. However, we believe this may be due to a floor effect since the difference yields RMSEs that are relative close in magnitude to the fixation + diameter input features from the 10 s output problem type.

While the information contained in embeddings is redundant with the fixation positions, we believe the categorical representation of continuous data (i.e., three AOIs from the large space of possible fixation coordinates) improved LSTM learning to yield lower error rates. In fact, architectures designed with characteristics of sparse data in mind during design tend to optimize faster and avoid local minima (Duchi et al., 2013).

### 4.3. Input Features

In our tested cases, we did not find significant improvements to our model after including saccade and blink sequences to fixation and pupil diameter inputs. We believe this may be because saccades and blinks are not related to pupil diameter in a task that requires focus such as in instructional video viewing. Despite a lack of human research related to our finding, we note animal research where microstimulation affected pupil dilation independently of saccades (Wang et al., 2012), highlighting the limited association of covert attention to pupil dilation. Because we partly sought a study of the minimum amount of eye tracking data required to accurately predict pupil diameter, our findings show that input features like saccades and blinks are not as critical as fixation and pupil diameter data when predicting future pupil diameter. We expect this finding to be helpful when focusing efforts for algorithms modeling pupillary mechanisms.

We note that our framework allows for prediction of other averages of eye tracking measures, such as fixation duration during the output region, blink rate, AOI-specific measures, etc. In addition, a framework such as ours allows for prediction of sequences of data—for example, fixation positions or pupil diameters. In fact, these types of problems mirror those faced in natural language processing, where deep learning, sequence models have performed relatively better than other linear or non-linear models for sequence outputs. Future work is required in applying this to viewing patterns.

### 4.4. Limitations

The primary limitation of our study is the lack of interpretability for the best-performing (LSTM) model, a common problem in deep learning studies. In this case, however, we attempted to solve the problem of not being able to understand the precise importance of input features by studying the effect of various models with modular inputs. We believe that this approach, paired with multiple runs of models to get average accuracy, addresses issues of interpretability and can be expanded upon in future work.

Additionally, we acknowledge that the LSTM model may be difficult to generalize to some training sequences. Our results on model accuracy, given modular inputs, allows some generalizability to sensors that are unable to extract pupil diameters or classification models unable to specify eye events such as saccades. However, a limitation of our approach is the lack of specificity of which LSTM hyperparameters or characteristics of eye events may be contributing to better accuracy of prediction. While our focus was on studying the effectiveness of RNNs in improving pupil prediction accuracy, and how student performance differences may be related to model accuracy, future work in this area should apply the same modularity within RNNs to further understand why deep learning models more effectively capture behavioral variations relative to their non-linear counterparts.

## 5. Conclusion

Our evaluation shows that, using AOI embeddings and fixation and pupil sequence history, a deep learning, sequence model predicts pupil diameter better than a naive mean-based estimate. Prediction is better for subjects who perform relatively poorly on post-lecture assessments, and model errors correlate with performance as a trend. This latter finding may indicate that those individuals were less engaged and thus had less expression of their cognition in their pupil dilation, allowing the model to capture luminance-influenced variations.

## Data Availability Statement

The raw data supporting the conclusions of this article will be made available by the authors, without undue reservation.

## Ethics Statement

The studies involving human participants were reviewed and approved by Columbia University Institutional Review Board. The patients/participants provided their written informed consent to participate in this study. Written informed consent was obtained from the individual(s) for the publication of any potentially identifiable images or data included in this article.

## Author Contributions

SK, LH, YM, KT, and PS conceived of the presented idea. SK developed the theory, performed the computations, and took the lead in writing the manuscript. PA provided data visualizations. LH and PS supervised the project. All authors provided critical feedback and helped shape the research, analysis, and manuscript.

## Conflict of Interest

SK and PA are founders at the company Fovea Inc. Fovea Inc. did not fund or take part in the experiment and analysis. The remaining authors declare that the research was conducted in the absence of any commercial or financial relationships that could be construed as a potential conflict of interest.
